# Partitioning and subsampling statistics in compartment-based quantification methods

**DOI:** 10.1371/journal.pone.0285784

**Published:** 2023-05-15

**Authors:** Manuel Loskyll, Daniel Podbiel, Andreas Guber, Jochen Hoffmann

**Affiliations:** 1 Advanced Technologies and Microsystems, Corporate Sector Research and Advance Engineering, Robert Bosch GmbH, Renningen, Baden-Württemberg, Germany; 2 Institute of Microstructure Technology (IMT), Karlsruhe Institute of Technology (KIT), Eggenstein-Leopoldshafen, Baden-Württemberg, Germany; 3 BioMEMS Consulting, Karlsruhe, Baden-Württemberg, Germany; Universitat de Vic - Universitat Central de Catalunya, SPAIN

## Abstract

The precision of compartment-based quantification methods is subject to multiple effects, of which partitioning and subsampling play a major role. Partitioning is the process of aliquoting the sample liquid and consequently the contained target molecules, whereas subsampling denotes the fact that usually only a portion of a sample is analyzed. In this work, we present a detailed statistical description comprising the effects of partitioning and subsampling on the relative uncertainty of the test result. We show that the state-of-the-art binomial model does not provide accurate results for the level of subsampling present when analyzing the nucleic acid content of single specific cells. Hence, in this work we address partitioning and subsampling effects separately and subsequently combine them to derive the relative uncertainty of a test system and compare it for single cell content analysis and body fluid analysis. In point-of-care test systems the area for partitioning and detection is usually limited, which means that a trade-off between the number of partitions (related to a partitioning uncertainty) and the amount of analyzed volume (related to a subsampling uncertainty) might be inevitable. In case of low target concentration, the subsampling uncertainty is dominant whereas for high target concentration, the partitioning uncertainty increases, and a larger number of partitions is beneficial to minimize the combined uncertainty. We show, that by minimizing the subsampling uncertainty in the test system, the quantification uncertainty of low target concentrations in single cell content analysis is much smaller than in body fluid analysis. In summary, the work provides the methodological basis for a profound statistical evaluation of partitioning and subsampling effects in compartment-based quantification methods and paves the way towards an improved design of future digital quantification devices for highly accurate molecular diagnostic analysis at the point-of-care.

## Introduction

Compartment-based absolute quantification, often implemented as digital polymerase chain reaction (dPCR), is an advancing method to quantify the amount of specific nucleic acid sequences in a sample liquid. This method is characterized by an aliquoting of the sample liquid into a large number of partitions, resulting in a distribution of the contained target sequence copies over these partitions. Each of the partitions serves as an independent reaction compartment for a nucleic acid amplification, usually using fluorescent probes as reporter for a successful amplification. Assuming ideal amplification, two populations of partitions can be separated based on the fluorescence signal after the amplification process: Partitions with an increased fluorescence signal (positive partitions) that initially contained at least one copy of the target sequence, therefor allowing a successful amplification reaction and partitions without increased fluorescence signal (negative partitions) that contain no copy after the distribution. Under the assumption that all partitions have the same volume and each copy has the same probability to be distributed to any of these partitions, the distribution of target copies can be described with Poisson statistics. The Poisson distribution P(k)=λkk!∙e−λ gives the probability for *k* target copies being in a partition if the mean copy load per partition equals *λ*. This equation simplifies for *k* = 0 resulting in *P*(*k* = 0) = *e*^−*λ*^. Since the ratio of positive partitions *R* can be approximately taken as 1−*P*(*k* = 0), the Poisson statistics provides the basis to estimate the mean copy load per partition *λ* by measuring the ratio of positive partitions *R* and calculating *λ* = -ln(1—*R*) [1]. By dividing the mean copy load per partition *λ* by the partition volume one can estimate the target copy concentration in the sample liquid. While quantitative real-time PCR (qPCR) has been the standard quantification method in molecular diagnostics ever since its development [2,3], dPCR has got more and more attention during the last years [4,5] facilitated by technological advances in microfluidic systems [6]. Especially its superior precision makes dPCR an attractive alternative to qPCR in various molecular diagnostic applications like the monitoring of low pathogenic loads [4,7], monitoring of minimal residual disease [8–10], the analysis of copy number variations [11] and the detection of rare mutants [12]. Over the last years dPCR systems have been extensively compared to qPCR systems for such use cases [13–16] confirming the enormous potential of this technology. The benefits of dPCR can also be exploited to investigate differences in nucleic acid content of single cells [17–20].

While the Poisson distribution allows giving an estimation of the concentration of a specific nucleic acid sequence based on the observed percentage of positive partitions, it does not provide information on the accuracy of this estimation. Since the target sequence copies are randomly distributed over the partitions, the observation of a given positive rate could originate from different target concentrations in the sample resulting in an uncertainty of the estimation which is called partitioning error. To judge the suitability of a certain quantification system for a given diagnostic application, it is crucial to assess the system’s quantification range and quantification precision. Consequently, Huggett et al. recommend to state the quantification uncertainty according to the *Minimum Information for the Publication of Digital PCR Experiments* [21,22].

The main sources of uncertainty described in literature comprise partitioning und subsampling errors, pipetting errors, volume variability between the partitions and misclassification of partitions [23,24]. Subsampling error occurs when not the whole sample but only a fraction of it is analyzed. This only allows an assessment of the target concentration in the analyzed fraction is not necessarily exactly the same as in the whole sample. While strategies to correct for the effects of volume variability [24,25] and misclassification [26–28] have been published, the partitioning and subsampling error can only be reduced by appropriate design. The state-of-the-art approach to determine the uncertainty caused by both these errors is based on modelling the amount of detected positive partitions as the result of a binomial process in which the success probability of a partition being positive equals the observed rate of positives *R*. The expected value of this binomial distribution then coincides with the number of observed positive partitions in the experiment. Still, other success probabilities in the binomial distribution might lead to the observed experimental result. Consequently, a confidence interval is constructed around the success probability of the binomial process to cover the desired confidence level. Since the success probability *R* for partitions being positive is directly linked to the mean copy load per partition *λ*, the confidence intervals are also directly connected to each other, as well as the confidence interval around the number of target copies in the sample [24,29–33]. One method to define the confidence interval around the success probability in a binomial distribution is the Clopper-Pearson confidence interval. However, we show that the results of this model of a binomial process for the combination of partitioning and subsampling error are only accurate if the subsampling effect is sufficiently high. Though, the analysis of the nucleic acid content of a single specific cell, for instance, the investigation of mRNA [19], miRNA [18] or mitochondrial DNA [20], subsampling does not occur during sample taking at the patient’s site but only after cell lysis inside the test system. Still, the partitioning process inside the test system might lead to some dead volume [34], especially if the combined volume of the partitions is smaller than the sample volume that is processed in the test system.

The presented approach is based on two distributions, each addressing either the partitioning or the subsampling effect separately. First, the description of the partitioning effect is derived combinatorically and then verified by Monte Carlo simulations in which the probability of a certain experimental outcome was simulated assuming that the copy load in the sample is known. By applying Bayes’ theorem, a so-called digitization distribution is derived which gives the probability for different numbers of target copies being in the sample if the amount of positive partitions is known. This distribution enables an analytical and exact calculation of the isolated partitioning uncertainty. Subsequently, the analytical description of the partitioning uncertainty is combined with a distribution accounting for the subsampling uncertainty to generate a combined distribution. This combined distribution deviates significantly from the state-of-the-art binomial model for low percentages of subsampling and low target concentrations while both models converge for large subsampling effects.

In quantification systems restricted to a limited evaluation area, the partition’s structure size defines the number of available partitions and the amount of sample that can be analyzed. In such systems, it is not possible to design the partitions in a way to minimize both partitioning uncertainty and subsampling uncertainty at the same time for a given processed sample volume. The combined distribution including both partitioning and subsampling effects provides the possibility to assess the contribution of these effects to the quantification uncertainty. In this work, an exemplary micro well-based quantification system is studied regarding the combined relative uncertainty caused by partitioning and subsampling for different partition diameters and target copy concentrations. To demonstrate how the combined distribution differs from the state-of-the-art binomial model if the overall level of subsampling is small, the results for body fluid analysis and single cell content analysis are compared. As it turns out, the minimal uncertainty attributed to partitioning and subsampling for low target concentrations can be much smaller in the latter than in the former if subsampling effects are minimized. As their high precision is a key feature of compartment-based quantification methods, the presented findings emphasize the suitability of these methods for the detection of rare targets in single cell content analysis.

## Materials and methods

### Monte Carlo simulations

For a verification of the analytically derived distribution of positives, the calculated expected values for different outcomes were compared to the results of Monte Carlo simulations. The Monte Carlo simulations were carried out using MathWorks Matlab R2019b. Typically, systems designed for compartment-based quantification comprise thousands or even millions of partitions [4,35]. The presented simulations however are limited to a set of 100 partitions to reduce the needed computational resources. In each simulation, a given number of copies was simulated to be randomly distributed over 100 partitions in 100,000 independent experiments. For this purpose, each copy was assigned to a random number between 1 and 100 with uniform probability representing the index of the partition the copy is allocated in. After all copies got a number assigned, the amount of numbers that occurred at least once, representing the positive partitions, was determined. The data points in the simulated distributions indicate how many of the 100,000 experiments showed the respective number of positive partitions. The data of the Monte Carlo simulations are generated with MC-simulation.m found in S1 File.

### Calculation of the confidence interval’s borders

When determining the border of the confidence interval with the digitzation statistic, the Stirling number of second kindgrows rapidly if the number of subsets is increased. Therefore, it is necessary to use a programming language capable of interpreting very large integer values for the computations. The shown data was generated by employing Python 3.7 on a Lenovo ThinkPad T470 (Intel® Core™ i5-6300U CPU @ 2.40 GHz, 8 GB RAM) to perform the calculations. Formula (7) allows to check whether a certain number of target copies *C* is covered by the confidence interval, but it does not provide a direct calculation of the upper and lower border of the confidence interval, *C*_U_ and *C*_*L*_, respectively. Therefore, a successive approximation using multiple iterations was performed for the determination of the borders. The approximation starts with an estimate *C*_est_ and a chosen initial step length *ΔC*_initial_ that is either added to the estimate if the inequality statement in Formula (7) is false or subtracted if it is true. Depending on the choices of *C*_est_ and *ΔC*_initial_ it can take multiple steps before the target value *C*_U_ (or *C*_L_) is crossed for the first time, which is indicated by a change of the Boolean value of the inequality statement. From that point onward the step length *ΔC* is halved and rounded to the nearest integer every time, until *ΔC* = 1. Accordingly, for this iteration process, it is convenient to choose the initial step length to a power of 2. In this way, it is possible to gradually approach the minimum (or maximum) value of *C* that fulfills the inequality and consequently represents the upper (or lower) bound of the confidence interval. The computation time is dependent on the quality of the initial guess *C*_est_ and how large *ΔC*_initial_ is chosen but is in general much lower than summing up all the probabilities that would be needed for a brute-force calculation using Formula (6).

A numerical issue is given by the fact that the elements of the sum can become very low, being interpreted as zero by the computation software if the constant factors are factored out. On the other hand, if nothing is factored out, the provided precision of both the “float” and “decimal” data format is not sufficient to accurately represent the fractions of the summation elements and deliver correct results. To avoid this issue, the constant factor (*N* - 1)! in the nominator was included during summation, while the constant factors in the denominator were excluded. Consequently, the elements of the summation become so large that they can be treated as integers instead of fractions resulting only in a loss of precision in the least significant digit. Since the result of the summation is divided by the factored out denominator with a very large value, this loss of precision is neglectable.

For the successive approximation of a specific mean copy load per partition the partitioning uncertainty can be calculated with PartitioningUncertainty_singleLambda.py which is provided in S1 File. If the partitioning uncertainty for all possible mean copy loads should be determined in a system, PartitioningUncertainty_allLambda.py automatically provides suiting *C*_est_ and *ΔC*_initial_.

The confidence interval of the binomial model is calculated by means of the Clopper-Pearson confidence interval. The Clopper-Pearson interval is an exact confidence interval around the success probability *p* of a binomial distribution. In the binomial model it is assumed that ratio of positive partition *R* is an approximation of the success probability *p* for a partition to be classified positive. Hence, the Clopper-Pearson confidence interval around *p* can be taken as confidence interval around *R* which in turn corresponds to a confidence interval around the mean copy load per partition *λ*. The relative uncertainty calculated from the confidence interval around *λ* is identical to the one calculated from the confidence interval around the number of copies C or the one around the target concentration because all these values can be transformed into one another by multiplication with a factor which cancels out in the calculation of the relative uncertainty.

## Results

### The digitization distribution and its relevance for partitioning uncertainty

#### Confidence interval and relative uncertainty

Due to the randomness of the copy distribution process, the measured ratio of positive partitions *p* generally does not coincide with the value that is predicted by Poisson statistics for a given mean load per partition *λ*. Consequently, the result of a digital quantification experiment deviates from the true value of nucleic acid target copies in the sample. However, statistical analysis allows to define a so-called confidence interval that contains the true value in (1-*α*) ∙ 100% of the time. The width of the confidence interval correlates with the uncertainty of the test result: A broad confidence interval indicates a high measurement uncertainty or poor measurement precision while a narrow confidence interval implies a low uncertainty and thus a high precision. Based on the lower and the upper bound of the confidence interval, *C*_L_ and *C*_U_ respectively, the relative uncertainty *σ* around the experimental result $\hat{C}$, which is the number of copies in the sample calculated using Poisson statistics, can be defined as

$$\sigma =\frac{\max(\left|\hat{C}‐C_{U}|,|\hat{C}‐C_{L}\right|)}{\hat{C}}.$$ (1)

In this work, the confidence interval is defined to cover the true value in 95% of the time (*α* = 0.05), with the true value being larger than the upper bound in 2.5% of the cases and lower than the lower bound in the remaining 2.5%. However, all shown methods can be adapted for other coverages of the confidence interval. Since the bounds are not necessarily symmetric around the estimator $\hat{C}$, the bound that gives the larger difference will define the uncertainty according to Formula (1).

The distribution *P*(*C*|*H*), called the digitization distribution, represents the conditional probability of *C* copies being in the sample if *H* positive partitions are observed. It allows calculating the bounds *C*_L_ and *C*_U_ of the (1-*α*) confidence interval thereby enabling the determination of the relative uncertainty *σ* of a digital quantification result according to Formula (1). The bounds are determined by a summation of the probabilities *P*(*C*|*H*) for a continuously increasing number of copies in the sample until the specified coverage is achieved:

$$\begin{array}{c} C_{L}=\max C^{*}|\sum_{C=H}^{C^{*}}P(C|H)\leq \frac{\alpha }{2}\\ C_{U}=\min C^{*}|\sum_{C=H}^{C^{*}}P(C|H)\geq 1‐\frac{\alpha }{2}. \end{array}$$ (2)

For an exact calculation of the confidence interval the digitization distribution *P*(*C*|*H*) will be derived in the next subsection.

#### Combinatorial derivation of the digitization distribution

In order to derive the digitization distribution *P*(*C*|*H*), we first have a close look at the partitioning process and the combinatorics behind it. The distribution process can be regarded as assigning each of the *C* copies, which are continuously numbered, one after another to one of the *N* distinguishable partitions. Given a certain amount of copies in the sample, there is a variety of ways to allocate these copies to specific partitions in a particular order. Since every single copy can end up in one of the *N* partitions, there are *N*^*C*^ particular orders of distributing *C* copies. Each of these allocation orders has the same probability of 1/*N*^*C*^ to occur, if the chance for a partition to be filled with a copy is equal for all partitions. Some of the allocation orders result in the same number *H* of partitions that contain at least one copy, consequently being classified as positive. Adding up the probabilities of these *O* allocation orders leading to the same number of positive partitions *H* allows to form a distribution P(H|C), referred to the distribution of positives. Since the probability for each single order is the same, the addition simplifies to a multiplication of the probability 1/*N*^*C*^ with the number of orders *O*. Combinatorial analysis leads to an accurate description of the size of this subset *O* of all possible allocation orders.

There are three aspects in which the allocation orders in the subset can differ from each other: the partitions showing a positive signal, the order in which the partitions become positive by getting their first copy assigned and lastly which copies end up together in a single positive partition. The first two aspects, the selection of *H* positive partitions out of *N* available partitions with consideration of the order of selection, can be described by a variation without repetition with the known value of *N*! ⁄ (*N* - *H*)!. The last aspect, the number of ways, in which *C* copies can end up in *H* positive partitions can be calculated by the Stirling number of second kind *S*(*C*, *H*). For example, S(3, 2) is 3, since {1}{2, 3}, {1, 2}{3} and {1, 3}{2} are the three possibilities to arrange three labeled copies into exactly two non-empty partitions. The order of the sets included by the Stirling number does not play a role here because it was taken into account by the variation without repetition already. Multiplying these three terms gives an expression for *O* and results in the distribution of positives

$$P(H|C)=O∙\frac{1}{N^{C}}=S(C,H)∙\frac{N!}{(N‐H)!}∙\frac{1}{N^{C}}$$ (3)

when also including the constant probability of 1/*N*^*C*^ for any of these allocation orders to occur. This expression the mathematically identical to the distribution P(H|C)=(NH)∑i=0H−1[(−1)i(HH−i)(H−i)C]NC Debski et. al used in their work to describe the partitioning and detection process [36].

**Fig 1:**
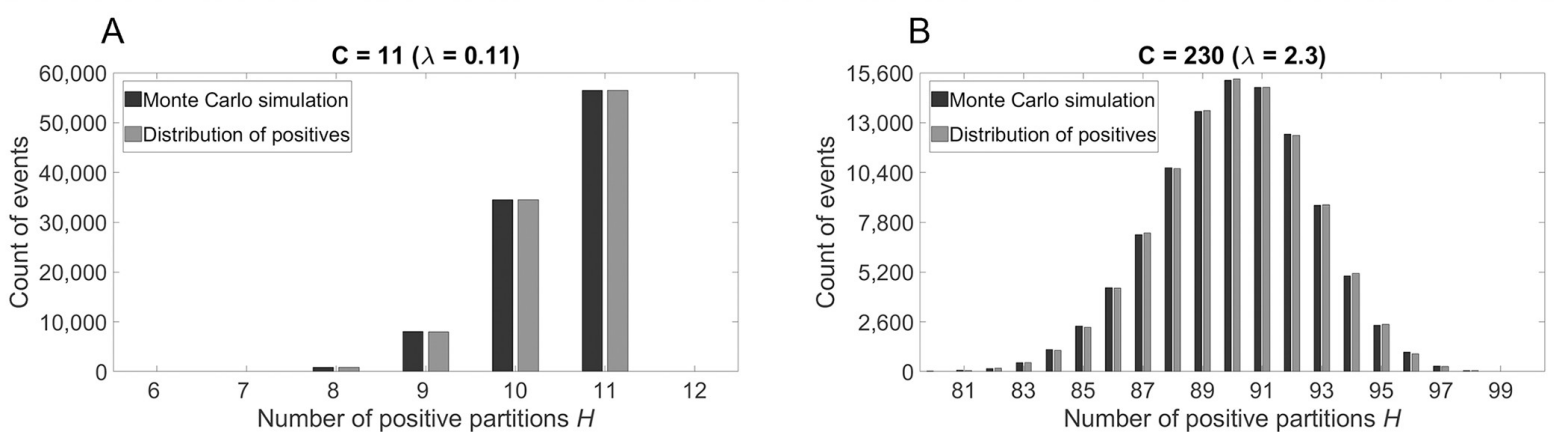
Comparison of the distribution of positives and the results of Monte Carlo simulations for *N* = 100 partitions. (A) Small number of contained copies *C* resulting in a low percentage of positive partitions; (B) High number of contained copies *C* resulting in a high percentage of positive partitions. The Monte Carlo simulations comprised 100,000 trials each and the amount of trials that result in a certain number of positive partitions was counted. The probabilities of the distributions are multiplied by the number of trials to obtain the expected values.

Fig 1 shows a comparison of the distribution of positives and a Monte Carlo simulation plotted for *N* = 100 partitions and a chosen number of copies of *C* = 11 (*λ* = *C* / *N* = 0.11) in panel (a) and *C* = 230 (*λ* = 2.3) in panel (b) corresponding to a low and a high percentage of positive partitions respectively. Additional data for other values of *C* is provided in S1 Fig. The distribution of positives matches the simulation tightly in all investigated cases.

A quantitative test result can only be derived for the case that at least one of the partitions remains negative, meaning it does not contain any target copy. The distribution of positives found stated in (3) enables to define an upper limit of quantification, a number of target copies *C* for which the probability of all partitions showing a positive signal is lower than a certain threshold value. For example, if there are *N* = 10,000 partitions available, then the probability of all of them being positives, resulting in failed quantification, exceeds 5% if the amount of copies in the sample is larger than *C* = 81,140. Consequently, the target load should not be increased above this value even though the theoretical dynamic range of the test goes up to about *C* = 92,100.

Moreover, the distribution of positives is very useful for the calculation of the partitioning uncertainty of compartment-based quantification methods, as it can be transformed into the digitization distribution using Bayes’ theorem *P*(*C*|*H*) ∙ *P*(*H*) = *P*(*H*|*C*) ∙ *P*(*C*). The probability *P*(*H*) is the sum of all probabilities that end up with the result of *H* positive partitions, no matter what number of copies *C* was distributed, and can be calculated with the distribution of positives. It is reasonable to assume the probability *P*(*C*) of *C* copies being in the analyzed sample to be a uniform distribution to get an unweighted summation

$$P(H)=\sum_{C=H}^{\infty }P(C)∙P(H|C)=P(C)∙\sum_{C=H}^{\infty }P(H|C).$$ (4)

Inserting Eq (4) into Bayes’ theorem results in the digitization distribution

$$P(C|H)=\frac{P(H|C)∙P(C)}{P(C)∙\sum_{C=H}^{\infty }P(H|C)}=\frac{S(C,H)∙\frac{N!}{(N‐H)!}∙\frac{1}{N^{C}}}{\sum_{C=H}^{\infty }S(C,H)∙\frac{N!}{(N‐H)!}∙\frac{1}{N^{C}}}=\frac{S(C,H)∙\frac{1}{N^{C}}}{\sum_{C=H}^{\infty }S(C,H)∙\frac{1}{N^{C}}}=\frac{1}{L}∙\frac{S(C,H)}{N^{C}}$$ (5)

with $L=\sum_{C=H}^{\infty }S(C,H)∙\frac{1}{N^{C}}=(N‐H‐1)!/(N‐1)!$ being the result of one of the generating functions of the Stirling number of second kind. The digitization distribution derived in (5) allows to evaluate compartment-based quantification experiments in more detail than the evaluation based on Poisson distribution could provide. It turns out that the result given by Poisson statistics is smaller than the statistical mean, but it is a very good estimation of the result that has the highest probability to occur. Furthermore, the digitization distribution enables to give exact bounds of the confidence interval therefore allowing an exact and analytical analysis of the uncertainty associated with the partitioning and detection process for the first time.

#### Calculation of confidence interval and relative uncertainty based on the digitization distribution

Now that the digitization distribution *P*(*C*|*H*) is derived, the condition formulated in (2) can be rewritten to

$$\begin{array}{c} C_{L}=\max C^{*}|\frac{(N‐1)!}{(N‐H‐1)!}∙\sum_{C=H}^{C^{*}}\frac{S(C,H)}{N^{C}}\leq \frac{\alpha }{2}\\ C_{U}=\min C^{*}|\frac{(N‐1)!}{(N‐H‐1)!}∙\sum_{C=H}^{C^{*}}\frac{S(C,H)}{N^{C}}\geq 1‐\frac{\alpha }{2}. \end{array}$$ (6)

It is sufficient to look at the upper confidence bound *C*_U_ as it turns out to give the larger deviation from the result $\hat{C}$ provided by Poisson statistics and consequently defines the relative uncertainty *σ* (compare Formula (1)). Hence, the shown calculation methods are primarily focused on finding *C*_U_ but could be adopted to find the lower confidence bound *C*_L_ as well.

To improve computational performance, Formula (6) is rewritten using the definition of the Stirling number and the characteristics of a geometric progression (detailed derivation is provided in S1 Appendix):

CL=maxC*|1(N‐H‐1)!∙H!∙NC∙∑j=0H(‐1)H‐j∙(Hj)∙jC+1∙(N‐1)!N‐j>1‐α2CU=minC*|1(N‐H‐1)!∙H!∙NC∙∑j=0H(‐1)H‐j∙(Hj)∙jC+1∙(N‐1)!N‐j<α2 (7)

The formulation in (7) allows to check if a certain *C* satisfies the condition of being inside the confidence interval. Nevertheless, the expression is in an implicit form which does not allow to calculate the bound of the confidence interval directly. For that reason, an iterative approximation algorithm was used for calculation, which is described in the methods section 2.2.

**Fig 2:**
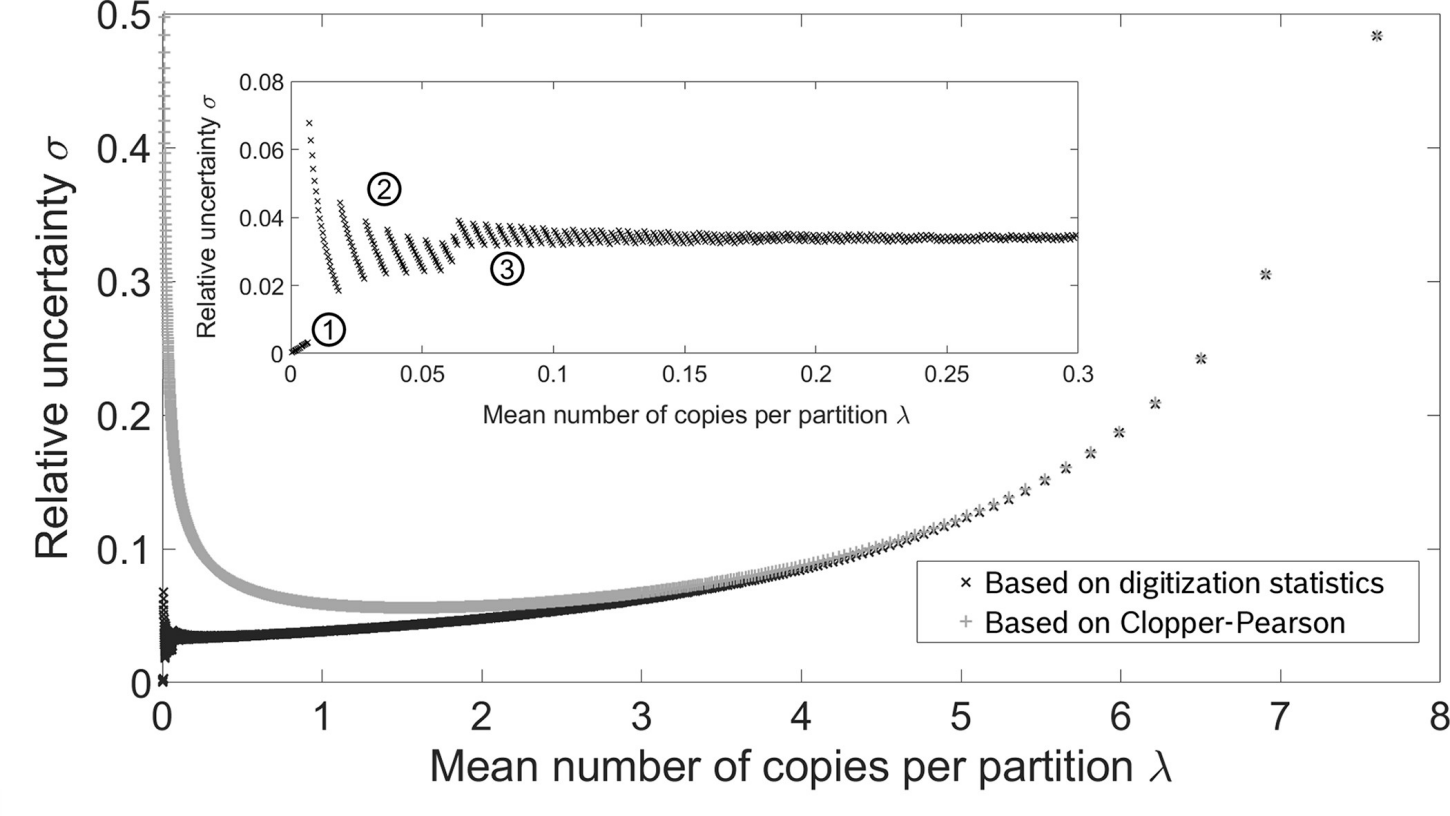
Relative uncertainty *σ* of a system with *N* = 2,000 partitions in dependence on the mean copy load per partition *λ*. The uncertainty was calculated using the digitization distribution (black) and the Clopper-Pearson interval that gives the confidence interval for the parameter *p* of a binomial distribution (grey). For large λ both approaches show very similar results. The inset depicts the relative uncertainty σ for *λ* < 0.3, where three phases can be distinguished: A counting phase (1), followed by a phase of a one-sided confidence interval (2) and eventually a phase of a two-sided confidence interval (3).

In Fig 2 the relative uncertainty (*α* = 0.05) based on the digitization distribution and the binomial distribution are plotted against the mean copy load per partition for a system with *N* = 2,000 partitions as indicated by the black and grey data points, respectively. The uncertainty associated with the binomial distribution is evaluated using the Clopper-Pearson interval. When comparing the confidence interval resulting from the digitization distribution and the Clopper-Pearson confidence interval, the Clopper-Pearson interval predicts a higher relative uncertainty if the mean copy number per partition *λ* is small, while the results of both calculation methods match better as *λ* is increased. For large mean copy loads however, the binomial distribution appropriately models the outcome of the classification process. Consequently, the assessment of the relative quantification uncertainty by means of the Clopper-Pearson confidence interval coincides with the results based on the digitization distribution in this case. Looking at the inset in Fig 2 one can distinguish three different phases: a counting phase (1), a phase with one-sided confidence interval (2) and the major phase of a two-sided confidence interval (3). In the counting phase (1), both the upper and lower bound *C*_L_ and *C*_U_ of the confidence interval are equal to the number of positive partitions *H*. Hence, the number of copies *C* is counted by the number of positive *H* with the desired confidence and Poisson statistics is not needed for the calculation of the result. In fact, in this phase the width of the confidence interval equals zero and a relative uncertainty of zero could be expected. Still, there is a small relative uncertainty as the experimental result $\hat{C}$ is slightly higher than the number of counted copies due to the correction related to Poisson statistics. For larger mean copy load *λ* the upper confidence bound *C*_U_ starts to increase above the number of positive partitions *H* while the lower bound *C*_L_ remains equal to *H*. Since it is impossible for the number of copies to be smaller than the number of positives *H*, the probabilities below the lower bound add up to zero resulting in a one-sided confidence interval (2). Consequently, no allowed value of *C* fulfills the condition in the first formula given in (6) and *α* can be set twice as large in the second formula, where the condition for of the upper bound formula is given, without changing the coverage of the confidence interval. As the lower confidence bound *C*_*L*_ also starts to be larger than the amount of positive partitions *H*, the graph enters the third phase, the two-sided confidence interval (3). In this phase the relative uncertainties calculated with the digitization statistics and the Clopper-Pearson confidence interval converge for larger values of the mean copy number per partition *λ*.

Due to the discrete nature of the digitization distribution, the relative uncertainty *σ* can only be calculated at certain data points and the bound of the confidence interval has to be an integer value. Accordingly, the relative uncertainty increases abruptly from time to time, when the width of the confidence interval is increased to include another integer value. This phenomenon can be seen in the inset of Fig 2 especially in the second phase and the beginning of the third phase. For the data points with a sufficiently large value of *λ* the effects of the discrete nature are less significant, and the digitization distribution predicts the relative uncertainty *σ* to keep increasing with larger mean copy load per partition *λ* approximately following a seventh-degree polynomial.

Based on further analysis an approximation method for the relative uncertainty for arbitrary numbers of partitions *N* and arbitrary mean copy loads per partition *λ* was developed which is described in S2 Appendix. It is exploiting the fact, that the relative uncertainty decays exponentially when increasing the number of available partitions, implying that a larger number of partitions reduces the relative uncertainty and thus improves the precision of a test result. Having said that, in a test system not only the partitioning uncertainty characterized by the digitization distribution, but also other effects contribute to the total quantification uncertainty. Apart from the partitioning uncertainty, the subsampling uncertainty another prominent effect in compartment-based digital quantification systems [30,32,37] that is effected by the partition design. Hence, the previously derived digitization distribution is combined with a subsampling distribution in the next section before drawing conclusions on how the partition size affects the precision of compartment-based digital quantification systems.

### Effect of partition size on the precision of digital quantification devices

#### Subsampling as source of uncertainty

The aliquoting of a sample into a large number of partitions is a characteristic process step in digital quantification, which is not needed when using other quantification approaches such as qPCR. Depending on the design and the aliquoting method, compartment-based digital quantification systems might have a certain dead volume, resulting in only a fraction of the loaded sample to be analyzed [34]. If the copies would always be evenly distributed over the sample volume, the ratio of copies in the analyzed sample to the copies in the loaded sample would coincide with the volume percentage of the analyzed sample. However, since the copies are randomly distributed in the sample, the number of copies in the analyzed fraction not always matches the expected value, resulting in a different concentration in the analyzed sample compared to the loaded sample. This leads to the so-called subsampling uncertainty since only the concentration in the analyzed partition can be measured and the concentration in the rest of the loaded sample remains unknown.

The binomial distribution P(C|Cload,p)=(CloadC)∙pC∙(1‐p)Cload‐C provides the probability that *C* copies are present in a given volume percentage *p* if *C*_load_ copies are contained in the loaded sample. This distribution *P*(*C*|*C*_load_, *p*) can be transformed into *P*(*C*_load_|*C*, *p*) by using Bayes’theorem. Again, analogously to the approach described in the derivation of the digitization distribution, a uniform distribution for *P*(*C*_load_) is assumed to get an unweighted summation. Further, the identity of $\sum_{C_{\mathrm{load}}=0}^{\infty }P(C|C_{\mathrm{load}},p)=1/p$, which results from one of the ordinary generating functions of the binomial coefficient, is used to obtain *P*(*C*_load_|*C*, *p*) in the form

P(Cload|C,p)=P(C|Cload,p)∙P(Cload)P(Cload)∙∑Cload=0∞P(C|Cload,p)=(CloadC)∙pC+1∙(1‐p)Cload‐C. (8)

Comparable to the digitization distribution, the subsampling distribution in Formula (8) allows the determination of the confidence interval by summation of the individual probabilities. Consequently, an assessment of the relative uncertainty associated with subsampling is possible. It is intuitive, that the uncertainty increases with a decreasing percentage *p* of the analyzed sample volume. As already has been described elsewhere [30,32], the subsampling uncertainty is more prevalent for smaller target copy concentrations. Regarding the influence of the partition size on the precision of compartment-based quantification devices, both sources of uncertainty–partitioning and subsampling–should be considered, since the true copy number *C* in the analyzed sample is never exactly known. The two distributions can be easily combined to

$$P(C_{\mathrm{load}}|H,p)=\sum_{C=H}^{C_{\mathrm{load}}}P(C_{\mathrm{load}}|C,p)∙P(C|H)$$ (9)

which allows the construction of a confidence interval for the estimated number of copies in the whole sample *C*_load_ based on the number of positive partitions *H* that have been observed.

The combined distribution in Formula (9) allows a detailed analysis but is very laborious in its computation. Debski et. al came to the conclusion, that the results of the combined distribution are equal to the outcome of a binomial distribution [36]. However, this is only the case if the mean copy load per partition is high (compare Fig 2) or the subsampling effect is sufficiently high, as is shown in S2 Fig. In single cell content analysis the subsampling effects are small in comparison to body fluid analysis and consequently a binomial model might not provide accurate results. Therefore, a multistep approximation is proposed for the calculation of the combined relative uncertainty of partitioning and subsampling effects in a compartment-based digital quantification device in the usecase of single cell content analysis. First, both the relative uncertainties due to partitioning and subsampling are estimated separately. In case of the uncertainty caused by the partitioning process, the approximation provided in S2 Appendix was used whereas for the subsampling uncertainty a different approximation presented in S3 Appendix was exploited. The two relative uncertainties of the partitioning and the subsampling process can be added in quadrature to estimate the combined relative uncertainty:

$$\sigma_{\mathrm{combined}}\approx \sqrt{{\sigma_{\mathrm{partitioning}}}^{2}+{\sigma_{\mathrm{subsampling}}}^{2}}.$$ (10)

The summation in quadrature of relative uncertainties is usually used in error propagation if two uncorrelated inputs are combined to an output value in a multiplication or division [38], but can also be used to combine errors in compartment-based digital quantification systems [32]. For the most cases, this approximation shows good accordance to the calculations based on the combined distribution and the quality of approximation improves for a larger number of available partitions *N* (compare S4 Appendix). The summation in quadrature with approximated inputs deviates more from the exact results but still gives a good approximation especially if the number of available partitions *N* and the number of positive partitions *H* are large. The Clopper-Pearson confidence interval on the other hand does only provide accurate results for low percentages *p* of analyzed sample indicating strong subsampling effects.

#### Design parameters of compartment-based quantification systems

Different approaches exist to realize the aliquoting of a sample liquid into partitions [23]. In all of them, the number of partitions and their combined volume is defined by certain design parameters. Hereinafter, we focus on micro well-based systems with hexagonal wells arranged in a honeycomb shape to get the densest packing, but the presented methods can also be applied to other designs as well.

**Fig 3:**
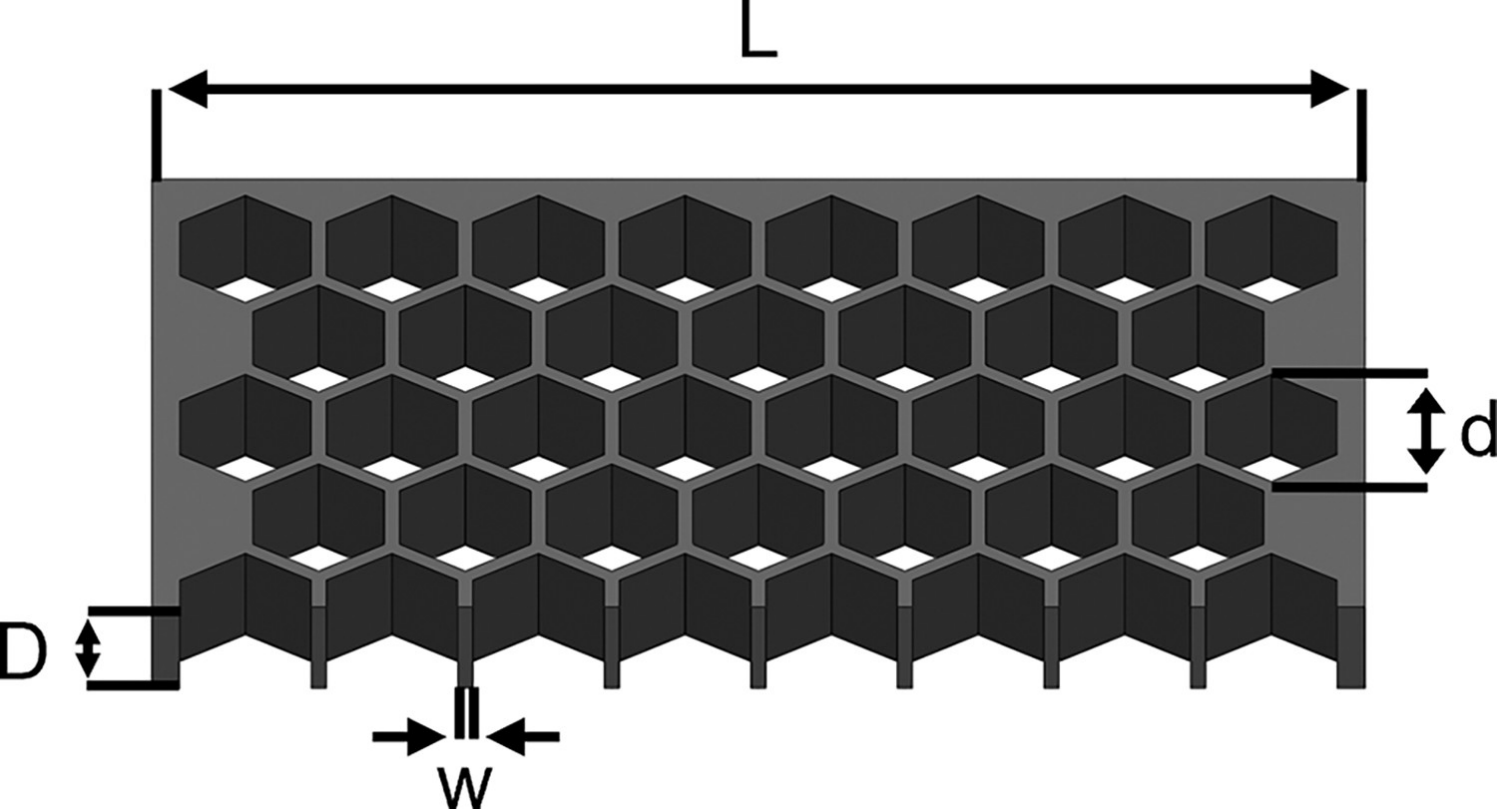
Schematic illustration of a micro well-based approach using hexagonally shaped compartments of outer diameter *d* that are arranged on a quadratic chip of side length *L* and thickness *D*. The hexagonal shape of the compartments allows the densest packing with a constant wall thickness *w* to all adjacent compartments. The side length *L*, the wall thickness *w* and the diameter of the partitions *d* limit the number of partitions on the chip. The volume of the partitions is defined by their hexagonal shape, the chip thickness *D* and the partition diameter *d*.

Diagnostic systems are usually limited in their size, so the area covered by the partitions may not exceed a certain area. This is especially true for point-of-care systems and systems with a parallel optical readout of all partition where the partitions must fit into the field of view of the detection optic. Fig 3 illustrates the design parameters that define how many partitions can be packed in the readout area of a micro well-based system. These parameters not only define the number of partitions, but also the volume that can be analyzed inside the partitions. For the sake of simplicity, the field for optical readout of the system is assumed to be quadratic with the side length *L*.

In a micro well-based setup, the outer diameter *d* of the hexagonally shaped partitions and the wall thickness *w* define the areal density of partitions, while the outer diameter *d* and the chip thickness *D* define the volume of each partition. In contrast, in a droplet-based system, the wall thickness would equal zero and the diameter *d* of the droplets would be sufficient to calculate the areal density of partitions and their volume. An important observation for all approaches is the fact that the number of partitions increases with smaller dimensions of the partitions, whereas the analyzed portion of the sample *V*_ana_ within a given area decreases. In systems, where walls separate the partitions, more partitions lead to more walls that take up some of the space leaving less area and consequently less volume for the liquid inside the partitions. Droplet-based systems have a wall thickness of zero, but in this case the spherical geometry of the partitions causes them to take up less volume if the diameter is decreased. This relation is crucial for the relevance of the two major sources of error: On the one hand, the subsampling error is reduced when a larger percentage *p* of the sample is analyzed, meaning that larger partitions would be favorable in order to reduce the subsampling uncertainty. On the other hand, however, the partitioning uncertainty is reduced when more partitions are available for partitioning, meaning that smaller partitions would are preferable to minimize the partitioning uncertainty. This results in contrary tendencies for both primary sources of uncertainty. Due to these contrary effects, one cannot state whether smaller or larger partitions result in a lower relative uncertainty of the result without further considerations. In fact, a detailed statistical analysis is required to investigate which effect dominates under which circumstances and if there exists a sweet spot that corresponds to an overall lowest relative uncertainty of the obtained quantification results.

#### Quantification accuracy of micro well-based systems

**Fig 4:**
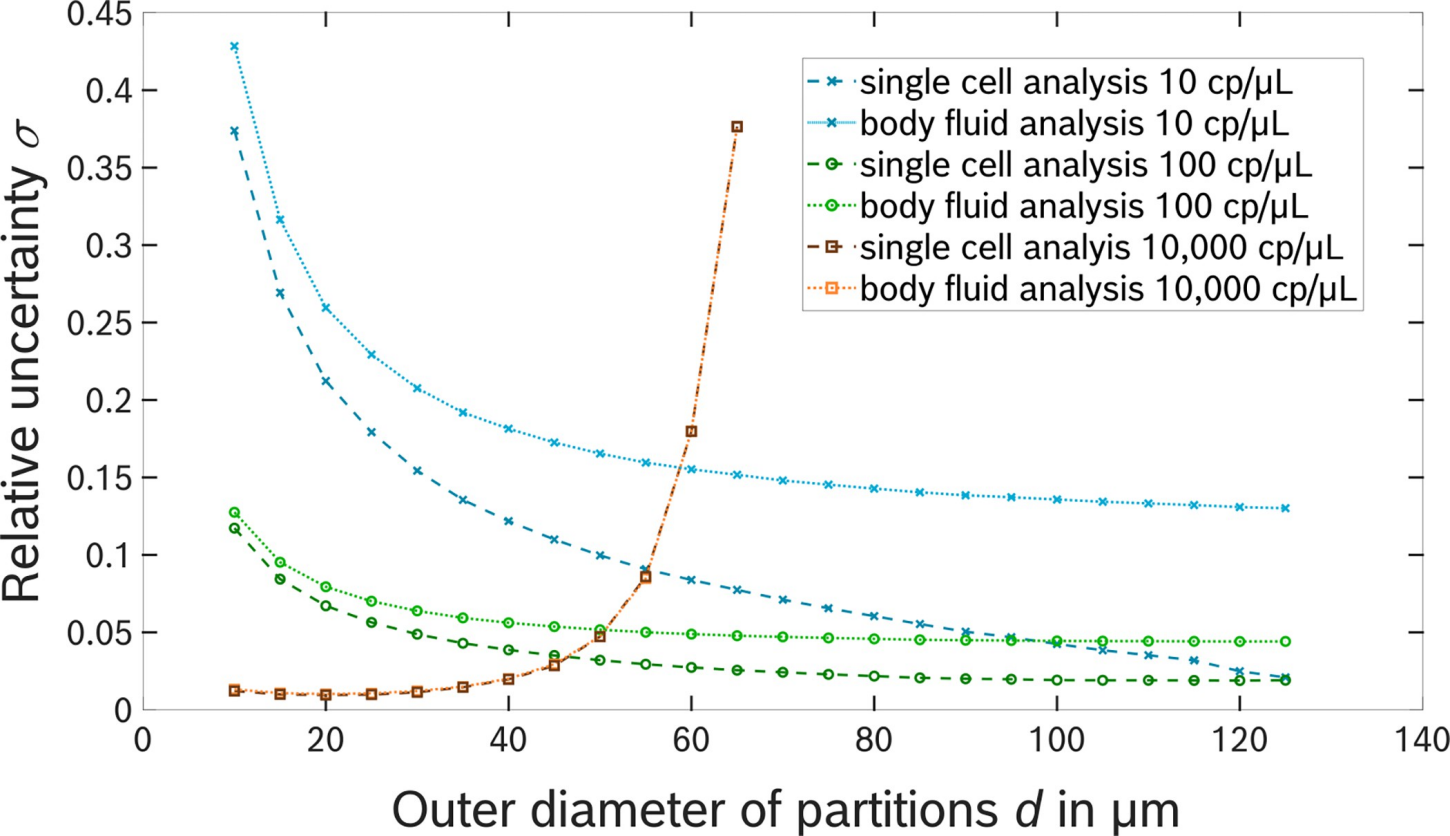
A plot of the relative uncertainty *σ* in digital quantification for a micro well-based system in dependence on the outer diameter *d* of the partitions for three different target concentrations of 10 cp/μL, 100 cp/μL and 10,000 cp/μL. In this example the chip was assumed to have a quadratic shape with side length of *L* = 10 mm and a thickness of *D* = 380 μm. The wall thickness was set to *w* = 25 μm and the system volume amounted to *V*_load_ = 25 μL. The data points for single cell content analysis calculated by the addition in quadrature of the partitioning and subsampling uncertainties are shown in black, whereas the data points calculated for body fluid analysis based on the Clopper-Pearson confidence interval are shown in grey. The curves show that the relative uncertainty achieved in compartment-based quantification devices for low target concentrations can be lower in single cell analysis than for the analysis of body fluids. For large target concentrations, the results for both applications converge and give similar results.

Subsequently, such a detailed statistical analysis is performed for a micro-well based system with hexagonally shaped partition arranged in a honeycomb pattern. In Fig 4 the use cases of single cell content analysis and body fluid analysis are compared regarding their overall relative uncertainty. As indicated earlier, the state-of-the-art binomial model does not provide accurate results for application in single cell content analysis. Consequently, while the data for body fluid analysis is calculated based on the Clopper-Pearson confidence interval, the data for single cell content analysis is generated using the previously introduced addition in quadrature. The python script Uncertainty_combinedErrors.py, which is applied for this calculation, is provided in S1 File.

The design parameters illustrated in Fig 3 correspond to *L* = 10 mm, *D* = 380 μm, *w* = 25 μm and the sample volume loaded to the system was assumed to be *V*_load_ = 25 μL. The minimum partition size considered in the analysis was set to 10 μm in this example, as this value represents a reasonable resolution limit of simple optics in point-of-care systems with a large field of view [39]. Data points are generated in 5 μm steps up to a partition diameter of *d =* 125 μm, as *d* = 127.4 μm is the smallest diameter that allows analysis of the entire loaded sample volume and further increasing the partition diameter any further would reduce neither partitioning nor subsampling uncertainty.

The relative uncertainty *σ* was calculated for four different target concentrations of 10 copies/μL (cp/μL), 100 cp/μL, 1,000 cp/μL (not shown in Fig 4) and 10,000 cp/μL. For a concentration of 10 cp/μL, the relative uncertainty *σ* calculated by the summation in quadrature decreases monotonically with the size of the partitions. The minimum in the uncertainty is reached for *d* = 127.4 μm resulting in *N* = 6,248 with a value of *σ* = 0.02 indicating that the relative uncertainty is dominated by the subsampling error in this case. This behavior can also be expected for smaller concentrations since the effect of subsampling increases when the concentration is decreased. In comparison, the Clopper-Pearson confidence interval results in higher values of the relative uncertainty *σ*, first decreasing with increasing partition diameter and then asymptotically approaching the value of *σ* = 0.13.

The curve for a concentration of 100 cp/μL also decline with rising partition diameter at first for both calculation methods, but this time the minimum is located at *d* = 119.1 μm and *N* = 6,975, while the uncertainty increases again slightly for larger partition sizes. Again, the Clopper-Pearson confidence interval leads to larger relative uncertainties than the summation in quadrature, while the difference is not as prominent as for the concentration of 10 cp/μL. The shift of the minimum relative uncertainty towards smaller partitions and thus a larger number of partitions can be attributed to the upcoming effect of the partitioning uncertainty and is more visible when the concentration is increased to 1,000 cp/μL and 10,000 cp/μL, where the minimum occurs at *d* = 49 μm (*N* = 25,308) and *d* = 21.5 μm (*N* = 60,685) respectively. As the concentration is increased, the results of provided by the two methods converge and are nearly identical for the concentration of 10,000 cp/μL. Due to the limitation in the maximum dynamic range, the number of partitions is not sufficient to provide a quantitative test result for the concentration of 10,000 cp/μL if the diameter of the partitions is larger than 65 μm. Eventually, the minimum in relative uncertainty is assumed to be obtained at the smallest considered partition diameter if the concentration is sufficiently high, as the subsampling uncertainty is negligible for high concentrations whereas the partitioning error is dominant in this scenario.

In summary, the regimes of low and high target concentration differ regarding the dependence of the relative uncertainty on the partition diameter. In case of a low target concentration, the minimal relative uncertainty is provided by a system capable of analyzing the entire loaded sample to avoid subsampling error. In this regime, applications in the field of single cell analysis profit even more from avoiding subsampling than use cases where a body fluid is analyzed. For high target concentrations the partitioning error is more prominent, and the relative uncertainty is minimized by increasing the number of available partitions *N* at the cost of increasing subsampling effects.

## Discussion and conclusion

The derived digitization distribution allows an analytical calculation of the uncertainty associated with the partitioning and detection process in compartment-based quantification devices for the first time. While Debski et al. already presented an approach addressing the uncertainty due to the partitioning and detection process, in their work they estimated the standard deviation and did not directly calculate the width of the confidence interval. Moreover, it was shown, that it is important to address the uncertainties caused by partitioning and subsampling independently if subsampling effects are small. This is especially the case when analyzing the nucleic acid content of single specific cells, where subsampling only occurs after cell lysis inside the test device but not during sample taking. It is already known, that subsampling effects are more prominent for small target concentrations but as it turns out the impact is even stronger in the analysis of single cell content. Consequently, by analyzing the entire available sample inside the test system and therefor eliminating subsampling effects, the relative uncertainty for low target concentration can be significantly reduced for these applications.

Even though an analytical calculation of the combined relative uncertainty caused both by partitioning and subsampling effects is presented and would be possible, an approximative approach was pursued due to computational effectiveness. While this approximation is not flawless, it shows close agreement with the exact data if the number of available partitions is large enough. The fact, that the data generated by the approximation converges with the relative uncertainty calculated by means of a binomial distribution for larger target concentrations, supports the quality of the approximation.

The overall quantification performance of a compartment-based system could be optimized for example by increasing the field of view of the optical system in order to use a larger area for analysis. A better resolution of the detection optics would allow smaller partitions, resulting in a wider possible dynamic range, but having no influence on the relative uncertainty in the quantification of low concentrations. Decreasing the wall thickness and increasing the chip thickness would enable the analysis of the same volume fraction in smaller and therefore more partitions, also lowering the overall relative uncertainty of the test system. However, due to limitations in the aspect ratio of the fabrication process, the chip thickness cannot be chosen too large, and the wall thickness should not drop below a certain value to ensure a reliable separation of the partitions.

We used a micro well-based system with arbitrary design parameters as an example for the application of the presented distributions. However, the described procedure can also be applied to droplet-based systems or all sorts of partitioning methods with arbitrarily chosen design parameters used for compartment-based quantification measurements. The results regarding the impact of the partition size on the relative uncertainty are especially relevant for systems that use a parallel readout of the partitions on a certain area. A serial fluorescence detection, for example of droplets flowing in a channel, may not show the same conflict in minimizing partitioning and subsampling uncertainty since the spatial constraints for the analysis are not given. Still, every test system can be assessed regarding its relative uncertainty when considering its number of available partitions and the percentage of analyzed sample. Furthermore, it is important to note that only the partitioning process and subsampling inside the system have been included into the calculation of the system’s relative uncertainty as these are directly influenced by the design of the partitions. For the sake of simplicity, the amplification was assumed to be ideal, excluding the effect of false-positives or false-negatives. Furthermore, variations in the partition volume were not considered even though it is reported that they have a significant influence on the quantification precision [12,40]. Beyond that, there might be additional sources of uncertainty upstream of the workflow, like pipetting errors or subsampling steps, whereas the presented analysis only covers the relative uncertainty of the diagnostic test system itself. For a comprehensive assessment of the uncertainty of a test result in practical application, all these sources of uncertainty have to be included.

In conclusion, the present work introduced the digitization distribution for a profound statistical evaluation of the partitioning uncertainty in compartment-based quantification methods. The combined analysis of partitioning and subsampling uncertainties emphasizes the importance of avoiding subsampling with a high sample transfer efficiency in single cell analysis and paves the way towards an improved design of future digital quantification devices for highly accurate molecular diagnostic analysis at the point-of-care in this field.

## Supporting information

S1 Fig Additional data providing a comparison of the distribution of positives and Monte Carlo simulations for *N* = 100 partitions. The values of *C* increase from panel (A) to panel (D) and result in a certain medium percentage of positive partitions. The Monte Carlo simulation consisted of 100,000 trials and the amount of trials that result in a certain number of positive partitions was counted. The probabilities of the distribution of positives is multiplied by the number of trials to get the expected values.
(TIF)

S2 Fig Percentage of analyzed sample above which the binomial distribution deviates significantly from the combined distribution for different mean copy loads per partition. If the percentage of analyzed sample is larger than the respective data points, then the probability at the expected value differs more than five percent between the binomial model and the combined distribution. The lower the mean copy load per partition, the higher subsampling effects must be for the binomial distribution to provide accurate results.
(TIF)

S1 File Matlab and Python scripts used for data generation. (ZIP)

S1 Appendix Derivation of the equation used in the successive approximation. (DOCX)

S2 Appendix Approximation of the relative uncertainty for arbitrary mean copy loads and number of available partitions. (DOCX)

S3 Appendix Approximation of the subsampling distribution with a Gaussian distribution. (DOCX)

S4 Appendix Comparison of the relative uncertainty calculated with the combined distribution, exact summation in quadrature, approximated summation in quadrature and based on Clopper-Pearson confidence interval for different number of partitions *N*, number of positives *H* and percentages of analyzed sample *p*. (DOCX)
