# Supplementary figures and images for: Partitioning and subsampling statistics in compartment-based quantification methods
Source: PLoS One. 2023 May 15;18(5):e0285784. doi: 10.1371/journal.pone.0285784 (PMC10184943; doi:10.1371/journal.pone.0285784)

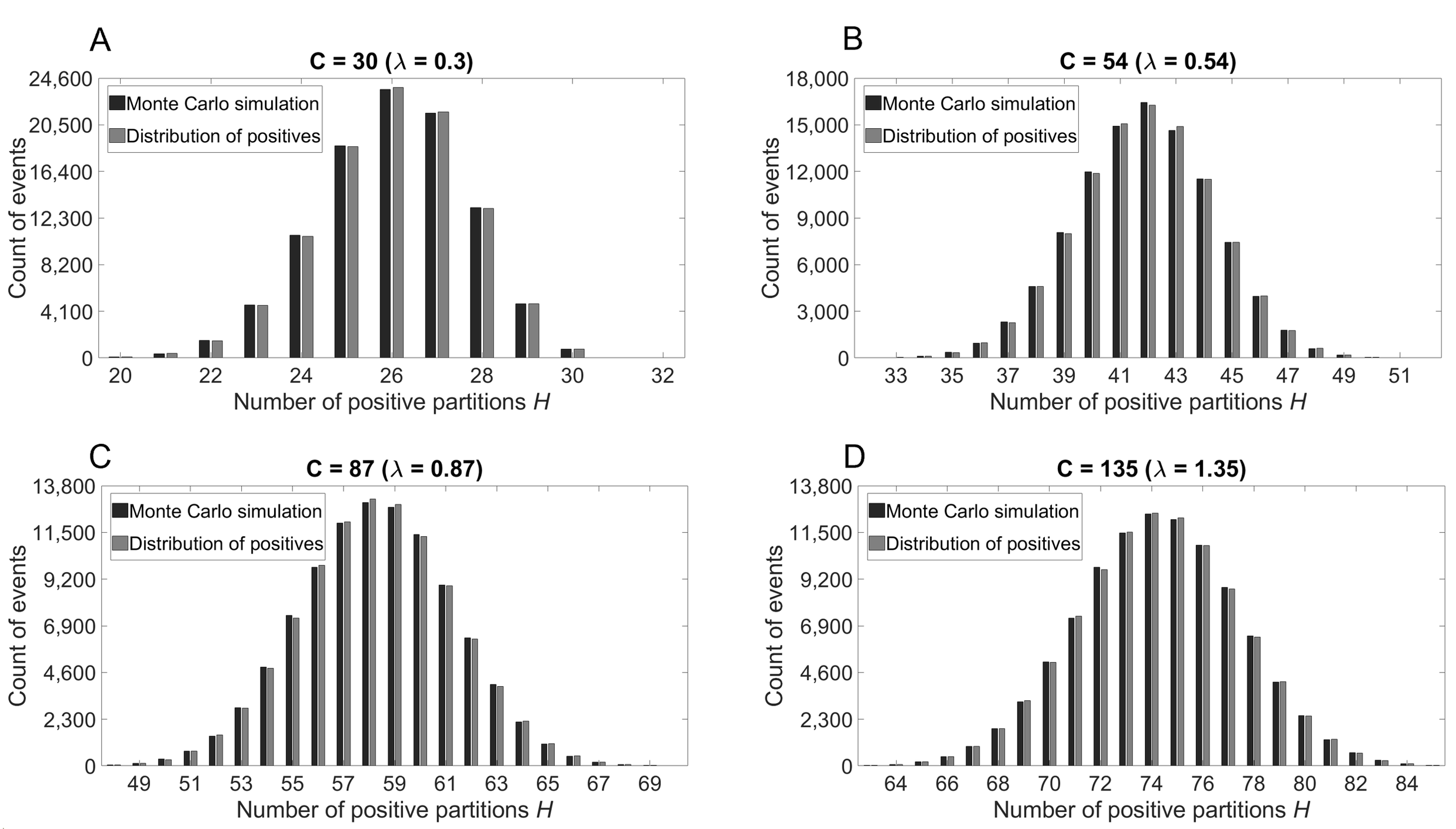

Supplement: S1 Fig — The values of C increase from panel (A) to panel (D) and result in a certain medium percentage of positive partitions. The Monte Carlo simulation consisted of 100,000 trials and the amount of trials that result in a certain number of positive partitions was counted. The probabilities of the distribution of positives is multiplied by the number of trials to get the expected values. (TIF) [file pone.0285784.s001.tif]

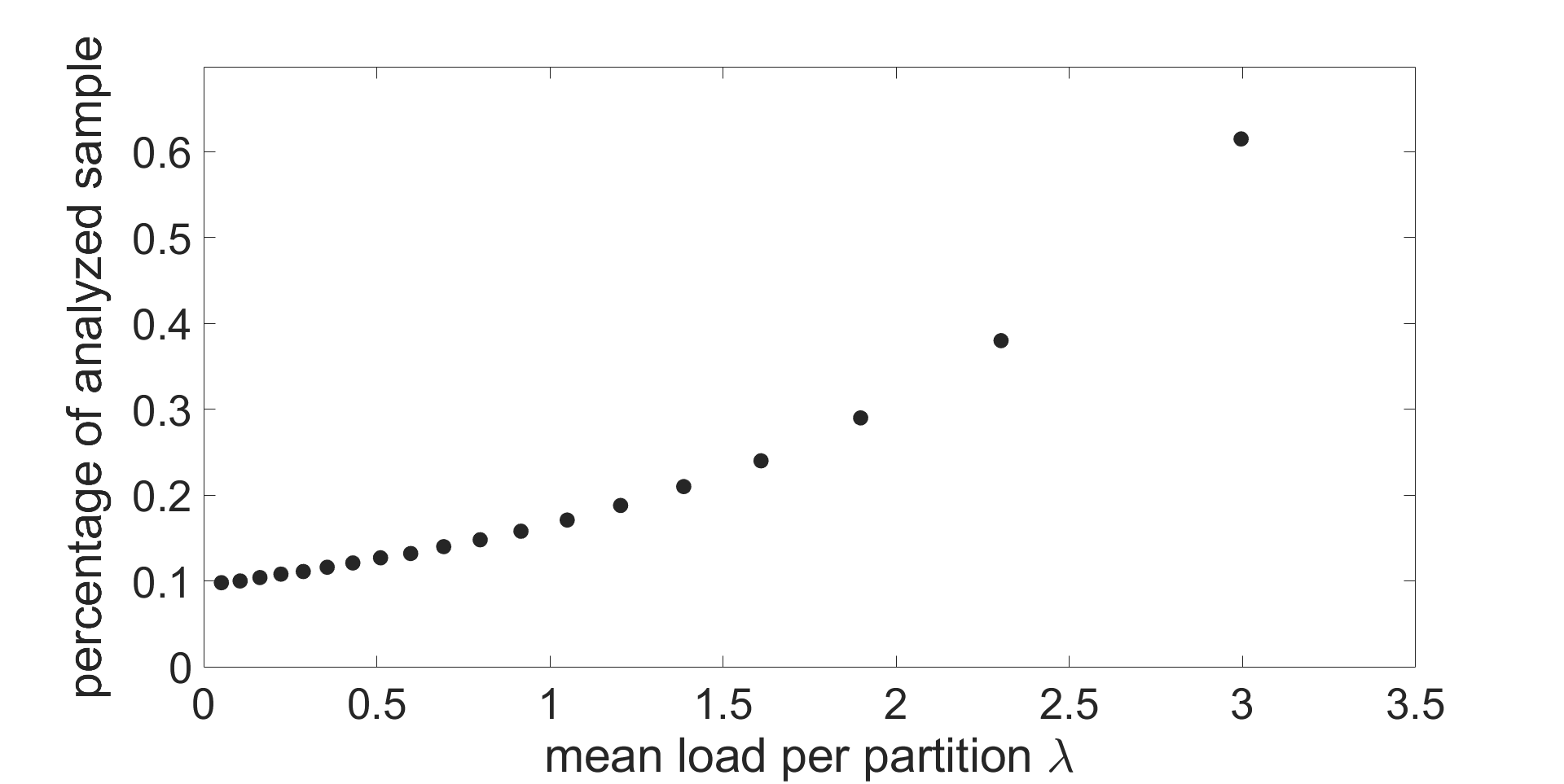

Supplement: S2 Fig — If the percentage of analyzed sample is larger than the respective data points, then the probability at the expected value differs more than five percent between the binomial model and the combined distribution. The lower the mean copy load per partition, the higher subsampling effects must be for the binomial distribution to provide accurate results. (TIF) [file pone.0285784.s002.tif]
