# Supplementary material for: Partitioning and subsampling statistics in compartment-based quantification methods
Source: PLoS One. 2023 May 15;18(5):e0285784. doi: 10.1371/journal.pone.0285784 (PMC10184943; doi:10.1371/journal.pone.0285784)
Supplement: S1 Appendix — (DOCX) [file pone.0285784.s004.docx]

**Derivation of the equation used in the successive approximation.**

By using the definition for the explicit form of the Stirling number of second kind and rearranging the sums one can write the condition for the borders of the confidence interval as

| $\frac{\left( \text{N}\text{ - 1} \right)\text{!}}{\left( \text{N}\text{ - }\text{H}\text{ - 1} \right)\text{!}}\text{ ∙}\sum_{\text{C}\text{ = }\text{H}}^{\text{C}^{\text{*}}} \frac{\text{S}\text{(}\text{C}\text{, }\text{H}\text{)}}{\text{N}^{\text{C}}}\text{ =} \frac{\text{1}}{\text{L}}\text{ ∙}\sum_{\text{C}\text{ = 0}}^{\text{C}^{\text{*}}} \frac{\text{1}}{\text{N}^{\text{C}}}\text{ ∙ }\text{S}\text{(}\text{C}\text{, }\text{H}\text{)}\text{}$ $\text{= }\frac{\text{1}}{\text{L}}\text{ ∙}\sum_{\text{C}\text{ = 0}}^{\text{C}^{\text{*}}} \left[ \frac{\text{1}}{\text{N}^{\text{C}}}\text{ ∙ }\frac{\text{1}}{\text{H}\text{!}}\text{ ∙}\sum_{\text{j}\text{ = 0}}^{\text{H}} \left( \text{-1} \right)^{\text{H}\text{ - }\text{j}}\text{ ∙ }\binom{\text{H}}{\text{j}}\text{ ∙ }\text{j}^{\text{C}} \right]$ $\text{= }\frac{\text{1}}{\text{L}}\text{ ∙ }\frac{\text{1}}{\text{H}\text{!}}\text{ ∙}\sum_{\text{j}\text{ = 0}}^{\text{H}} \left[ \left( \text{-1} \right)^{\text{H}\text{ - }\text{j}}\text{ ∙ }\binom{\text{H}}{\text{j}}\text{ ∙}\sum_{\text{C}\text{ = 0}}^{\text{C}^{\text{*}}} \left( \frac{\text{j}}{\text{N}} \right)^{\text{C}} \right]$ $\text{= }\frac{\text{1}}{\text{L}}\text{ ∙ }\frac{\text{1}}{\text{H}\text{!}}\text{ ∙}\sum_{\text{j}\text{ = 0}}^{\text{H}} \left( \text{-1} \right)^{\text{H}\text{ - }\text{j}}\text{ ∙ }\binom{\text{H}}{\text{j}}\text{ ∙ }\text{s}_{\text{C}}\text{.}$ | (1) |
| --- | --- |

The inner sum, which is denoted as $\text{s}_{\text{C}}$, represents a partial sum of a geometric series and has the known value

| $\text{ }\text{s}_{\text{n}}\text{ =}\sum_{\text{k}\text{ = 0}}^{\text{n}} \text{q}^{\text{k}}\text{ = }\frac{\text{1 - }\text{q}^{\text{n}\text{ + 1}}}{\text{1 - }\text{q}}\text{.}$ | (2) |
| --- | --- |

Combining (1) with (2) and rearranging the terms gives

| $\frac{\left( \text{N}\text{ - 1} \right)\text{!}}{\left( \text{N}\text{ - }\text{H}\text{ - 1} \right)\text{!}}\text{ ∙}\sum_{\text{C}\text{ = }\text{H}}^{\text{C}^{\text{*}}} \frac{\text{S}\text{(}\text{C}\text{, }\text{H}\text{)}}{\text{N}^{\text{C}}}\text{ =} \frac{\text{1}}{\text{L}}\text{ ∙ }\frac{\text{1}}{\text{H}\text{!}}\text{ ∙}\sum_{\text{j}\text{ = 0}}^{\text{H}} \left( \text{-1} \right)^{\text{H}\text{ - }\text{j}}\text{ ∙ }\binom{\text{H}}{\text{j}}\text{ ∙ }\text{s}_{\text{C}}\text{}$ $\text{= }\frac{\text{1}}{\text{L}}\text{ ∙ }\frac{\text{1}}{\text{H}\text{!}}\text{ ∙}\sum_{\text{j}\text{ = 0}}^{\text{H}} \text{(-1)}^{\text{H}\text{ - }\text{j}}\text{ ∙ }\binom{\text{H}}{\text{j}}\text{ ∙ }\frac{\text{1 - }{\text{(}\frac{\text{j}}{\text{N}}\text{)}}^{\text{C}\text{ + 1}}}{\text{1 - }\frac{\text{j}}{\text{N}}}$ $\text{= }\frac{\text{1}}{\text{L}}\text{ ∙ }\left( \frac{\text{1}}{\text{H}\text{!}}\text{ ∙}\sum_{\text{j}\text{ = 0}}^{\text{H}} \left( \text{-1} \right)^{\text{H}\text{ - }\text{j}}\text{ ∙ }\binom{\text{H}}{\text{j}}\text{ ∙ }\frac{\text{1}}{\text{1-}\frac{\text{j}}{\text{N}}} \text{- }\frac{\text{1}}{\text{H}\text{!}}\text{ ∙}\sum_{\text{j}\text{ = 0}}^{\text{H}} \left( \text{-1} \right)^{\text{H}\text{ - }\text{j}}\text{ ∙ }\binom{\text{H}}{\text{j}}\text{ ∙ }\frac{{\text{(}\frac{\text{j}}{\text{N}}\text{)}}^{\text{C}\text{ + 1}}}{\text{1 - }\frac{\text{j}}{\text{N}}} \right)$ $\text{= }\frac{\text{1}}{\text{L}}\text{ ∙ }\left( \text{M}\text{ - }\text{K} \right).$ | (3) |
| --- | --- |

Looking at the minuend M one can find the expression for the limit of the geometric series, which is

| $\text{ }\frac{\text{1}}{\text{1 - }\text{q}}\text{ =}\sum_{\text{k}\text{ = 0}}^{\text{∞}} \text{q}^{\text{k}}\text{= }\text{s}_{\text{∞}}\text{.}$ | (4) |
| --- | --- |

Using this identity and rearranging the summation for the minuend M while the subtrahend K remains untouched leads to

| $\frac{\left( \text{N}\text{ - 1} \right)\text{!}}{\left( \text{N}\text{ - }\text{H}\text{ - 1} \right)\text{!}} \text{∙}\sum_{\text{C}\text{ = }\text{H}}^{\text{C}^{\text{*}}} \frac{\text{S}\text{(}\text{C}\text{, }\text{H}\text{)}}{\text{N}^{\text{C}}}\text{ = }\frac{\text{1}}{\text{L}}\text{ ∙ }\left( \text{M}\text{ - }\text{K} \right)\text{}$ $\text{= }\frac{\text{1}}{\text{L}}\text{ ∙ }\left( \frac{\text{1}}{\text{H}\text{!}}\text{ ∙}\sum_{\text{j}\text{ = 0}}^{\text{H}} \left( \text{-1} \right)^{\text{H}\text{ - }\text{j}}\text{ ∙ }\binom{\text{H}}{\text{j}}\text{ ∙ }\frac{\text{1}}{\text{1 - }\frac{\text{j}}{\text{N}}} \text{- }\text{K} \right)$ $\text{= }\frac{\text{1}}{\text{L}}\text{ ∙ }\left( \frac{\text{1}}{\text{H}\text{!}}\text{ ∙}\sum_{\text{j }\text{= 0}}^{\text{H}} \left( \text{-1} \right)^{\text{H}\text{ - }\text{j}}\text{ ∙ }\binom{\text{H}}{\text{j}}\text{ ∙}\sum_{\text{C}\text{ = 0}}^{\text{∞}} \left( \frac{\text{j}}{\text{N}} \right)^{\text{C}}\text{- }\text{K} \right)$ $\text{= }\frac{\text{1}}{\text{L}}\text{ ∙ }\left( \sum_{\text{C}\text{ = 0}}^{\text{∞}} \frac{\text{1}}{\text{N}^{\text{C}}}\text{ ∙ }\frac{\text{1}}{\text{H}\text{!}}\text{ ∙ }\sum_{\text{j}\text{ = 0}}^{\text{H}} \left( \text{-1} \right)^{\text{H}\text{ - }\text{j}}\text{ ∙ }\binom{\text{H}}{\text{j}}\text{ ∙ }\text{j}^{\text{C}}\text{- }\text{K} \right)$ $\text{= }\frac{\text{1}}{\text{L}}\text{ ∙ }\left( \sum_{\text{C}\text{ = 0}}^{\text{∞}} \text{S}\text{(}\text{C}\text{, }\text{H}\text{) }\text{∙ }\frac{\text{1}}{\text{N}^{\text{C}}} \text{- }\text{K} \right)$ $\text{= 1 - }\frac{\text{K}}{\text{L}}.$ | (5) |
| --- | --- |

Now the defining equation for the upper bound of the confidence interval can be rewritten as

| ${\text{ }\text{C}}_{\text{U}}\text{ =}\min\text{C}^{\text{*}}\left\vert\frac{\left( \text{N}\text{ - 1} \right)\text{!}}{\left( \text{N}\text{ - }\text{H}\text{ - 1} \right)\text{!}}\text{ ∙}\sum_{\text{C}\text{ = }\text{H}}^{\text{C}^{\text{*}}} \frac{\text{S}\left( \text{C}\text{, }\text{H} \right)}{\text{N}^{\text{C}}}\text{ ≥ 1 - }\frac{\text{α}}{\text{2}} \right.\text{}$ $\text{=}\min\text{C}\left\vert\text{1 - }\frac{\text{K}}{\text{L}}\text{ ≥ 1 - }\frac{\text{α}}{\text{2}} \right.$ $\text{=}\min\text{C}\left\vert\frac{\text{K}}{\text{L}}\text{ < }\frac{\text{α}}{\text{2}} \right.$ $\text{=}\min\text{C}\left\vert\frac{\left( N - 1 \right)!}{\text{(N - H - 1)!}\text{ ∙ }\text{H}\text{!}}\text{ ∙}\sum_{\text{j}\text{ = 0}}^{\text{H}} \left( \text{-1} \right)^{\text{H}\text{ - }\text{j}}\text{ ∙ }\binom{\text{H}}{\text{j}}\text{ ∙ }\frac{{\text{(}\frac{\text{j}}{\text{N}}\text{)}}^{\text{C}\text{ + 1}}}{\text{1 - }\frac{\text{j}}{\text{N}}} \right.\text{ < }\frac{\text{α}}{\text{2}}$ $\text{=}\min\text{C}\left\vert\frac{1}{\text{(N - H - 1)!}\text{ ∙ }\text{H}\text{! ∙ }\text{N}^{\text{C}}}\text{ ∙}\sum_{\text{j}\text{ = 0}}^{\text{H}} \left( \text{-1} \right)^{\text{H}\text{ - }\text{j}}\text{ ∙ }\binom{\text{H}}{\text{j}}\text{ ∙ }\frac{\text{j}^{\text{C}\text{ + 1}}\text{ ∙ }\left( \text{N}\text{ - 1} \right)\text{!}}{\text{N}\text{ - }\text{j}} \right.\text{ < }\frac{\text{α}}{\text{2}}$ | (6) |
| --- | --- |

which results in the equation used in the successive approximation.
