# Supplementary material for: Partitioning and subsampling statistics in compartment-based quantification methods
Source: PLoS One. 2023 May 15;18(5):e0285784. doi: 10.1371/journal.pone.0285784 (PMC10184943; doi:10.1371/journal.pone.0285784)
Supplement: S2 Appendix — (DOCX) [file pone.0285784.s005.docx]

**Approximation of the relative uncertainty for arbitrary mean copy loads and number of available partitions.**

Deeper analysis of the results given by the digitization distribution reveals that the dependency of *σ*(*N*) on the number of available partitions for fixed *λ* can be approximated by an exponentially decaying relation of the form $\text{σ}\text{ ≈ }\text{a}\text{ ∙ }\text{N}^{\text{b}}$, as shown in Fig 1 for *λ* ≈ 3.22.


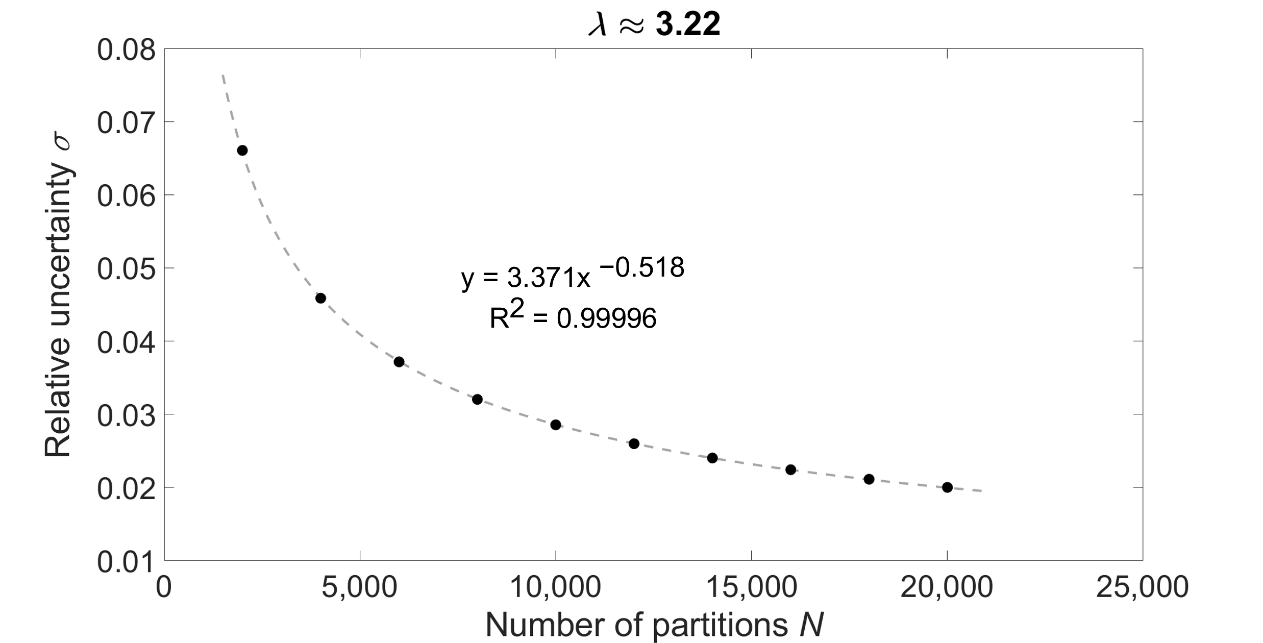


**Fig 1. Relative uncertainty *σ* in dependency of the number of partitions *N* for *λ* ≈ 3.22.**The uncertainty σ was determined by means of the digitization distribution for *N* = 2,000 up to N = 20,000 with increments of 2,000. An exponential function in the form$\text{σ }\text{= }\text{a }\text{∙ }\text{N}^{\text{ b}}$ was fitted to the calculated data. The high coefficient of determination *R*² = 0.99997 indicates a good agreement of the exponential approximation to the calculated values for *a* = 3.371 and *b* = -0.518.

The coefficient *a* and the exponent *b* have been determined for five values of *λ* between $\lambda_{1}\text{ ≈ 0.58}$ and $\lambda_{5}\text{ ≈ 3.91}$ by calculating the exact relative uncertainty σ over a range from *N* = 2,000 – 20,000 partitions for these specific values of λ and fitting an exponential function. The respective values of the fitting parameters are listed in Table 1.

**Table 1.** **Fitting parameters *a* and *b* of the exponentially decaying behavior of the relative uncertainty *σ* in dependence on the number of available partitions *N* for different mean copy loads per partition *λ*.**

| **Mean number of copies per partition *λ*** | **Coefficient *a*** | **Exponent *b*** |
| --- | --- | --- |
| 0.58 | 1.744 | - 0.512 |
| 1.27 | 1.936 | ‑ 0.509 |
| 2.12 | 2.396 | - 0.512 |
| 3.22 | 3.371 | - 0.518 |
| 3.91 | 4.416 | - 0.525 |

According to the exponential dependency, it is possible to estimate the relative uncertainty *σ* for the respective values of *λ* and an arbitrary amount of available partitions *N* by using the known coefficient *a* and the exponent *b*. These values can serve as grid points between $\lambda_{1}\text{ ≈ 0.58}$ and $\lambda_{5}\text{ ≈ 3.91}$ for the approximation of *σ*(*λ*) with the previously mentioned seventh-degree polynomial. Since the possible values of *λ* exceed the last grid point at $\lambda_{5}\text{ ≈ 3.91}$ for larger numbers of available partitions *N,* additional grid points are required to improve the quality of the fit. As the calculation of the relative uncertainty *σ* by the Clopper-Pearson interval shows good agreement with the results based on the digitization distribution when *λ* is large, it provides five additional grid points for the five largest possible values of *λ*. Using the Clopper-Pearson interval instead of the digitization distribution significantly reduces the computation time while giving almost the same result.

In Fig 2. an example of the seventh-degree polynomial approximation of *σ*(*λ*) for *N* = 10,000 is shown in comparison to the exact values that were calculated by means of the digitization distribution. The approximation shows good accordance with the data points, slightly underestimating the relative uncertainty for *λ* < 1 and slightly overestimating it for *λ* > 5.


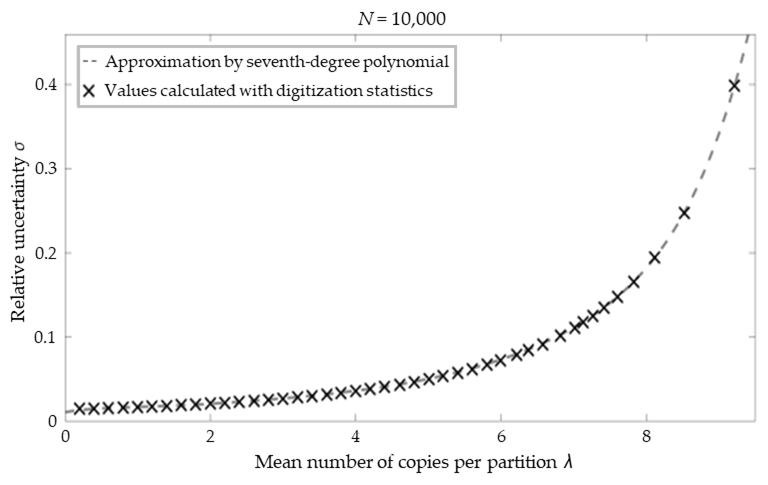


**Fig 2. Example for the approximation of the relative uncertainty *σ* for arbitrary values of *N* and *λ*.**The values of *σ* have been determined at some grid points ($\text{λ}_{\text{1}}$ ≈ 0.58, $\text{λ}_{\text{2}}$  ≈ 1.27, $\text{λ}_{\text{3}}$ ≈ 2.12, $\text{λ}_{\text{4}}$ ≈ 3.22, $\text{λ}_{\text{5}}$ ≈ 3.91) using the exponential dependency of *σ*(*N*). Due to the good agreement of the relative uncertainty calculated based on the Clopper-Pearson interval with the calculation based on the digitization statistics when *λ* is large, it was used for the five largest values of *λ* to provide five additional grid points. After fitting a seventh-grade polynomial function to the ten grid points, the approximation shows good accordance to the data calculated with the digitization statistics.
