# Supplementary material for: Partitioning and subsampling statistics in compartment-based quantification methods
Source: PLoS One. 2023 May 15;18(5):e0285784. doi: 10.1371/journal.pone.0285784 (PMC10184943; doi:10.1371/journal.pone.0285784)
Supplement: S3 Appendix — (DOCX) [file pone.0285784.s006.docx]

**Approximation of the subsampling distribution with a Gaussian distribution.**

The subsampling distribution *P* ($\text{C}_{\text{load}}$|*C, p*) was approximated by a Gaussian distribution, which allows a simplified calculation of the bounds of the confidence interval. A Gaussian is characterized by its mean value *μ* and its variance *s*. The probability at the mean value *μ*, where the Gaussian distribution reaches its maximum, is solely dependent on the variance *s*, since$\text{P}\left( \text{µ} \right)\text{ = }\frac{\text{1}}{\sqrt{\text{2π}\text{s}}}$. Consequently, knowing the peak probability of a distribution that is approximated by a Gaussian distribution is sufficient to estimate its variance *s*. The most probable result given by the digitization distribution based on the number of positive partitions *H* is assumed to represent the number of copies in the analyzed sample and is calculated by$\hat{\text{C}}\text{=}\left\lfloor\text{-}\log\text{(1 - }\frac{\text{H}}{\text{N}}\text{)}\text{ ∙ }\text{N} \right\rfloor$. This results in${\hat{\text{C}}}_{\text{load}}\text{ =}\left\lfloor\text{-}\log\text{(1 - }\frac{\text{H}}{\text{N}}\text{)}\text{ ∙ }\frac{\text{N}}{\text{p}} \right\rfloor$ being the most probable number of copies $\text{C}_{\text{load}}$ in the loaded sample according to the subsampling distribution. Hence, after calculating *P*(${\hat{\text{C}}}_{\text{load}}$|$\hat{\text{C}}$, *p*), which is equivalent to *P*(*µ*) in this case, the variance *s* of the subsampling distribution can be derived by exploiting the relation between *s* and *P*(*µ*) and the 95 %-confidence interval can be defined as ${\hat{\text{C}}}_{\text{load}}\text{ ± 1.96 ∙ }\sqrt{\text{s}}$. The relative uncertainty attributed to subsampling can be calculated by using the bounds of this confidence interval.
