# Supplementary material for: Partitioning and subsampling statistics in compartment-based quantification methods
Source: PLoS One. 2023 May 15;18(5):e0285784. doi: 10.1371/journal.pone.0285784 (PMC10184943; doi:10.1371/journal.pone.0285784)
Supplement: S4 Appendix — (DOCX) [file pone.0285784.s007.docx]

**Comparison of the relative uncertainty calculated with the combined distribution, exact summation in quadrature, approximated summation in quadrature and based on Clopper-Pearson confidence interval for different number of partitions N, number of positives H and percentages of analyzed sample p.**

Since the combined distribution need a long time to calculate for large numbers of available partitions *N*, data was only computed up to *N*= 300. For such a low number of available partitions the fitting parameters for the partitioning uncertainty provided in S4 Appendix do not give accurate results. Consequently, more suitable parameters listed in Table 1 were chosen for the comparison of the different approximation strategies to the uncertainty calculated with the combined distribution.

A comparison of the relative uncertainty calculated with the combined distribution, exact summation in quadrature, approximated summation in quadrature and based on Clopper-Pearson confidence interval for different number of partitions *N*, number of positives *H* and percentages of analyzed sample *p* is presented in Table 2. For the exact summation in quadrature, the exact partitioning uncertainty and the subsampling uncertainty were calculated with the digitization statistic and the modified binomial distribution respectively whereas for the approximated summation in quadrature the approximations presented in S4 and S6 were used respectively.

**Table 1.** **Fitting parameters *a* and *b* of the exponentially decaying behavior of the relative uncertainty *σ* in dependence on the number of available partitions *N* for different mean copy loads per partition *λ*.**

| **Mean number of copies per partition *λ*** | **Coefficient *a*** | **Exponent *b*** |
| --- | --- | --- |
| 0.223 | 1.577 | - 0.507 |
| 0.357 | 1.696 | ‑ 0.513 |
| 0.511 | 1.604 | - 0.505 |
| 0.693 | 1.706 | - 0.508 |
| 0.916 | 1.828 | - 0.511 |
| 1.204 | 1.910 | - 0.509 |
| 1.609 | 2.088 | - 0.510 |

**Table 2:** **Comparison of the relative uncertainty calculated with the combined distribution, exact summation in quadrature, approximated summation in quadrature and based on Clopper-Pearson confidence interval for different number of partitions *N*, number of positives *H* and percentages of analyzed sample *p*.**

| *N* | *H* | *p* | *σ* combined distribution | *σ* exact summation in quadrature | *σ* approximated summation in quadrature | *σ* Clopper-Pearson |
| --- | --- | --- | --- | --- | --- | --- |
| 100 | 10 | 0.1 | 0.793 | 0.814 | 0.593 | 0.718 |
|  |  | 0.5 | 0.611 | 0.598 | 0.468 | 0.718 |
|  |  | 0.9 | 0.282 | 0.289 | 0.256 | 0.718 |
|  | 50 | 0.1 | 0.316 | 0.309 | 0.278 | 0.312 |
|  |  | 0.5 | 0.263 | 0.258 | 0.236 | 0.312 |
|  |  | 0.9 | 0.195 | 0.197 | 0.183 | 0.312 |
|  | 90 | 0.1 | 0.306 | 0.302 | 0.298 | 0.305 |
|  |  | 0.5 | 0.290 | 0.289 | 0.287 | 0.305 |
|  |  | 0.9 | 0.274 | 0.276 | 0.275 | 0.305 |
| 200 | 20 | 0.1 | 0.514 | 0.514 | 0.421 | 0.488 |
|  |  | 0.5 | 0.376 | 0.361 | 0.323 | 0.488 |
|  |  | 0.9 | 0.196 | 0.180 | 0.173 | 0.488 |
|  | 100 | 0.1 | 0.214 | 0.212 | 0.196 | 0.214 |
|  |  | 0.5 | 0.179 | 0.176 | 0.166 | 0.214 |
|  |  | 0.9 | 0.136 | 0.137 | 0.128 | 0.214 |
|  | 180 | 0.1 | 0.204 | 0.201 | 0.196 | 0.204 |
|  |  | 0.5 | 0.193 | 0.191 | 0.187 | 0.204 |
|  |  | 0.9 | 0.182 | 0.182 | 0.178 | 0.204 |
| 300 | 30 | 0.1 | 0.405 | 0.408 | 0.342 | 0.391 |
|  |  | 0.5 | 0.297 | 0.294 | 0.265 | 0.391 |
|  |  | 0.9 | 0.168 | 0.149 | 0.152 | 0.391 |
|  | 150 | 0.1 | 0.171 | 0.168 | 0.160 | 0.173 |
|  |  | 0.5 | 0.142 | 0.138 | 0.135 | 0.173 |
|  |  | 0.9 | 0.108 | 0.106 | 0.105 | 0.173 |
|  | 270 | 0.1 | 0.161 | 0.160 | 0.156 | 0.163 |
|  |  | 0.5 | 0.154 | 0.152 | 0.149 | 0.163 |
|  |  | 0.9 | 0.145 | 0.144 | 0.141 | 0.163 |
